# A case–control study on the individualized use of opioid analgesics based on single-nucleotide polymorphism

**DOI:** 10.3389/fphar.2025.1629866

**Published:** 2025-08-12

**Authors:** Jing Yang, Lei Zheng, Fu Zheng, Shu-Han Zhou, Liang-Wei Sun, Chao Song

**Affiliations:** ^1^ Department of Pharmacy, Shandong Provincial Third Hospital, Cheeloo College of Medicine, Shandong University, Jinan, China; ^2^ Department of Pharmacy, Shandong Medical College, Jinan, China; ^3^ School of Medicine and Pharmacy, Ocean University of China, Qingdao, China

**Keywords:** opioid analgesics, single-nucleotide polymorphism, adverse drug reaction, drug dose demand, personalized medication

## Abstract

**Objective:**

The aim of this study is to explore the relationship between gene polymorphism and the efficacy and adverse drug reactions (ADRs) of opioid analgesics, improve medication safety, and promote personalized medication.

**Methods:**

First, the method of evidence-based medicine retrieval and evaluation was adopted to systematically summarize the opioid analgesic drug ADR and efficacy-related gene loci currently recommended by guidelines, authoritative genetic information databases, and low-bias clinical trial studies. The gene loci with strong correlation evaluation were selected as target genes for testing. Then, a clinical case–control trial was designed, divided into two groups: the ADR research group and the dose demand research group. A total of 480 enrolled patients were tested for target genes. Finally, the gene test results were evaluated for ADR association and drug dose association using SPSS 26.0 software and the SNPStats tool.

**Results:**

Four genes were selected for detection: *CYP2D6*, CYP3A5*3, *ABCB1*, and *OPRM1*. The results of the correlation analysis showed that, in the ADR research group, compared with the control subgroup, the AG + GG type distribution of *OPRM1* (rs1799971, A>G) was lower, and the AA type distribution was higher in the observation subgroup (*P* < 0.05). Individual statistics of patients using oxycodone also revealed differences in *OPRM1* (rs1799971, A>G). In particular, in the observation subgroup, the AA type accounted for 56.35% and the AG + GG type accounted for 43.65%; in the control subgroup, the AA type accounted for 38.28% and the AG + GG type accounted for 61.72%; compared with the control subgroup, the proportion of *OPRM1* (rs1799971, A>G) AA type was higher in the experimental subgroup (*P* < 0.05). The SNPStats program was used to analyze the association between single-nucleotide polymorphism (SNP) and ADR in five genetic models. The results showed that co-dominant, dominant, and allele models all suggested a significant association between *OPRM1* (rs1799971) and ADR incidence (*P* < 0.05). In the dose demand evaluation group, the results showed that there was no significant difference in genotype distribution between the observation subgroup (high-dose group) and the control subgroup (low-dose group) (*P* > 0.05). However, attention should be paid to the *ABCB1* gene. At the current sample size, the *ABCB1* (rs1045642, C>T) CC + CT ratio in the observation subgroup tends to be higher than that in the control subgroup compared to TT.

**Conclusion:**

*OPRM1* (rs1799971) AA genotype patients have a higher risk of ADR. *OPRM1* AA genotype patients should pay more attention to ADR, and the dosage can be initiated at low levels. The association between dose demand and SNPs still needs to be evaluated in larger cohorts. Meanwhile, particular attention should be given to the *ABCB1* gene.

## 1 Introduction

Opioid analgesics are mainly used to relieve moderate to severe pain. Although opioid analgesics play an important role in pain treatment, their clinical application currently faces many challenges. In clinical practice, it is found that the analgesic effect of opioid analgesics varies greatly among individuals. Patients with the same basic situation and pain assessment use the same dose of drugs, but some patients have insufficient analgesia or adverse drug reactions (ADRs), such as nausea, vomiting, urinary retention, constipation, and respiratory depression (Palada et al., 2018). Another common issue is the development of tolerance and dependence associated with opioid analgesics. Clinically, there are cases of drug tolerance caused by rapidly increasing the dosage, which also increases the risk of drug abuse (Jarvis et al., 2018). Therefore, promoting the precise use of opioid analgesics and achieving personalized medication may help address the clinical problems mentioned above and improve outcomes for pain patients, especially those suffering from cancer-related pain (Yan et al., 2018). Based on this clinical issue, we conducted precision medication application research on opioid analgesics based on single-nucleotide polymorphism (SNP), aiming to identify key gene loci that affect analgesic efficacy and toxic side effects, develop personalized medication plans, improve treatment efficacy and safety, and reduce drug abuse problems.

## 2 Methods

### 2.1 Screening target gene loci

We conducted a comprehensive search of published pharmacogenomic research on the clinical application related to the metabolism, transport, and analgesic targets of opioid analgesics; the specific search scope included the following: CPIC guidelines, genetic information databases (PharmGKB, Table of Pharmacogenomic Biomarkers in Drug Labeling, and SNPedia database), the PubMed database, and Chinese literature databases (such as CNKI). We collected published pharmacogenomic research evidence on the *in vivo* transport, drug metabolism, and analgesic-related targets of opioid analgesics and systematically summarized and screened gene loci related to the efficacy and toxic side effects of opioid analgesics. After evaluating and screening the data, the importance of different gene loci was evaluated based on PharmGKB’s pharmacogenomic clinical evidence strength scoring system to screen out target genes with strong correlation.

### 2.2 Research grouping and inclusion/exclusion criteria

The research subjects are cancer pain patients who used opioid analgesics at a tertiary hospital in Shandong Province, China, from September 2021 to March 2024. The study was approved and filed following a review by the hospital’s medical ethics committee (filing number: KYLL-20211115). The study was divided into the ADR research group and the dose demand research group, with safety and drug dose demand research conducted in each group. To ensure baseline consistency, a 1:1 screening was adopted for enrollment. The inclusion and exclusion criteria for each group are as follows.

ADR research group: The inclusion criteria for the observation subgroup (patients who experienced ADR) were as follows: ① age range of 18–85 years; ② cancer pain patients who only use opioid analgesics for pain relief have an initial NRS score of 4–10; and ③ occurrence of ADR after using opioid analgesics. Exclusion criteria were as follows: ① individuals with a history of mental or neurological disorders; ② patients with unclear consciousness or in a coma; ③ combined use of other types of analgesic drugs including combination preparations; ④ long-term use of glucocorticoids; and ⑤ individuals with a history of narcotic drug abuse or allergies to opioid analgesics. The inclusion criteria for the control subgroup (non ADR group) were as follows: ① age range of 18–85 years; ② cancer patients who only use opioid analgesics for pain relief have an initial NRS score of 4–10; and ③ those who did not experience ADRs after using opioid analgesics. The exclusion criteria are the same as above.

Dose demand research group: The Edmonton classification system sets the grading criteria for oral morphine as follows: low dose < 60 mg/d, medium dose 60–300 mg/d, and high dose > 300 mg/d. However, in clinical practice, except for a few advanced cancer patients, there are relatively few patients with a daily dose greater than 300 mg. Therefore, considering the sample size, the dose range is divided into oral morphine ≥150 mg and <150 mg.

The inclusion criteria for the observation subgroup (high-dose group) were as follows: ① age range of 18–85 years; ② cancer patients who only use opioid analgesics for pain relief have an initial NRS score of 4–10; ③ the daily dose of the drug (calculated as oral morphine) is ≥150 mg; and ④ no serious adverse drug reactions have occurred. Exclusion criteria were as follows: ① individuals with a history of mental or neurological disorders; ② patients with unclear consciousness or in a coma; ③ combined use of other types of analgesic drugs including combination preparations; ④ long-term use of glucocorticoids; and ⑤ individuals with a history of narcotic drug abuse or allergies to opioid analgesics. The inclusion criteria for the control subgroup (low-dose group) were as follows: ① age range of 18–85 years; ② cancer patients who only use opioid analgesics for pain relief have an initial NRS score of 4–10; ③ the daily dose of the drug (calculated as morphine) is less than 150 mg; and ④ no serious adverse drug reactions have occurred. The exclusion criteria are the same as above.

### 2.3 ADR evaluation method

We used the Karch–Lasagna method, which is commonly applied both domestically and internationally, to evaluate the causal relationship of ADRs and accurately identify those caused by opioid analgesics (Liu, 2023). The evaluation results are classified as follows: positive (the time sequence since medication is reasonable; the reaction is the same as the known ADR type of the drug, or the adverse reaction disappears after discontinuation and reappears after readministration); very likely (adverse reactions may occur after the patient takes the medication and cannot be explained by other diseases the patient is suffering from. The reaction disappears after discontinuation, but the reaction is different from the known ADR type of the drug or has not been repeated); and probably (conditional and suspicious). ADR types are divided into general and severe. Severe ADR refers to one of the following situations: causing death; carcinogenesis, teratogenicity, and birth defects; being dangerous to life and capable of causing permanent or significant disability to the human body; causing permanent damage to organ function; and causing hospitalization or prolonged hospital stay.

In addition to obtaining basic patient information and medication status through the electronic medical record system for ADR evaluation and medication efficacy evaluation, a patient information collection form is used to inquire with patients and their families before sampling to further determine the actual medication dosage, pain control status, ADR presentation type, severity, and outcome of the patient. Patients whose causal evaluation results of ADR are positive or highly likely to be included in the observation subgroup of the ADR research group are selected. The dose demand research group needs to exclude cases of patients with severe ADRs.

### 2.4 Genetic testing methods

SNP analysis was performed using fluorescence *in situ* hybridization (FISH) or polymerase chain reaction (PCR). To reduce trauma through non-invasive sampling and increase patient acceptance, the FISH method uses oral exfoliated cells as a substitute for blood samples. The method for detecting oral exfoliated cells is as follows: ① after rinsing the mouth thrice with water, the mucosa on both sides of the oral cavity is scraped using a specialized throat swab (30 times on each side) to collect oral exfoliated cells. ② The swab is placed back into the swab tube, taking care not to let it come into contact with any other person or object during this process. The collected oral swabs can be temporarily refrigerated but should not exceed 7 days. ③ A volume of 400 μl of the CQ-ENH type sample extract (mainly composed of 0.74% ammonium chloride solution) is slowly added into the throat swab tube. ④ The solution is thoroughly shaken and mixed. After standing at room temperature for 1 min, the extract is transferred to a 1.5-mL centrifuge tube. ⑤ It is centrifuged at 12,000 r/min for 5 min, and the supernatant is then prepared for inspection. ⑥ A measure of 1.0 μl of the upper-layer sample solution (3–4 mm below the liquid level) is taken and added to the wall of the detection reagent tube for testing on the machine. Samples can be processed and used immediately (within 1 h) and cannot be used beyond the time limit. ⑦ The FISH method uses double Z-shaped probes, which hybridize with the target sequence through the tandem connection of two independent Z-shaped probes. Then, the signal amplification precursor sequence can bind to the 28 bases in the upstream region of the double Z-shaped probe, resulting in a change in the stereo configuration of the final sequence and amplification of the signal. ⑧ The signals are recorded, and the detection site information is output.

*CYP2D6* (rs1065852, rs1135822, rs16947, rs28371725, and rs28371735) was detected via PCR. In particular, the total volume of the PCR reaction is 5 μL, including 0.5 μL of 10 × PCR buffer, 0.4 μL of 25 mmol/L MgCl_2_, 0.1 μL of 25 mmol/L dNTP mix, 1 μL of 0.5 μmol/L specific amplification primer mix, 0.2 μL of 5 U/μL PCR enzyme, 0.8 μL of ddH_2_O, and 2 μL of DNA template. The PCR amplification procedure is as follows: preheating at 95 °C for 2 min, followed by 35 cycles at 95 °C for 30 s, 56 °C for 30 s, and 72 °C for 1 min; 72 °C for 5 min and maintained at 4 °C. The primer sequence is as follows: rs1065852: F: GCC​CAT​TTG​GTA​GTG​AGG​CAG​GTA and R: AGC​AGT​ATG​GTG​TGT​TCT​GGA​AGT​C; rs1135822: F: CTCCAGCGACTTCTTGCC and R: GCT​AAT​GCC​TTC​ATG​GCC​ACG; rs16947: F: GTC​ACC​ATC​CCG​GCA​GAG​A and R: CCG​TTC​TGT​CCC​GAG​TAT​GC; rs28371725: F: GGG​TGT​CCC​AGC​AAA​GTT​CAT and R: CCG​TTC​TGT​CCC​GAG​TAT​GC; rs28371725: F: GGG​TGT​CCC​AGC​AAA​GTT​CAT and R: GAT​CCT​ACA​TCC​GGA​TGT​GCA​G; and rs28371735: F: GCA​CAG​CAC​AAA​GCT​CAT​AGG​G and R: CAC​TTC​AGC​TTC​TCG​GTG​CC.

Experimental instruments and reagents: a multi-channel fluorescence quantitative analyzer (FasCan 48S, Shanxi Medical Equipment Injection Standard No. 20182400043); a real-time fluorescence quantitative PCR instrument (7500 model, Applied Biosystems, United States); and detection reagents purchased from Guangyin Medical Technology Co., Ltd.

### 2.5 Statistical method

Statistical analysis was conducted using SPSS 26.0 software, and chi-square tests were used to analyze genotype and allele frequencies. The Hardy–Weinberg equilibrium (HWE) was assessed using the HWE program; a *P*-value > 0.05 indicates that the data are consistent with a population in genetic equilibrium, suggesting that the sample comes from the same Mendelian population. Further correlation analysis was conducted on the detection results using the SNPStats genetic model (https://www.snpstats.net/snpstats/start.htm). In the measurement data, if it follows a normal distribution and is represented by x̄ ± s, the t-test should be performed for intergroup comparison. If it is a skewed distribution, the median (minimum, maximum) [M (xmin, xmax)] is used to represent it, and the rank-sum test is performed for intergroup comparison. Count data are represented as n (%), and comparison between groups is performed using the chi-square test. If *P* < 0.05, it is considered that there is a significant statistical difference between the two sets of data.

## 3 Results

### 3.1 Determination of the target gene

Nine related genes were initially retrieved, and finally, four target genes related to ADR and drug efficacy were identified from gene loci recommended by a CPIC rating of B or above, a PharmGKB rating of 3 or above, and low-bias risk studies. The correlation between the gene and the ADR or efficacy is shown in Table 1.

**TABLE 1:** Information on genes to be tested.

Genotype/locus	ADR and drug efficacy correlation	Main drug involved	PharmGKB evidence level
*CYP2D6* (rs1065852, rs1135822, rs16947, rs28371725, and rs28371735)	ADR correlation: ultra-fast metabolizers (activity score > 2.25) should not use codeine or tramadol as they are prone to serious toxicity risks (Crews et al., 2021)	Codeine Tramadol	1A
	Dose-related: individuals with CYP2D6 metabolic disorders (activity score 0) should avoid using codeine and tramadol; intermediate metabolizers (activity score 0.25–1) should use the recommended dosage in the instruction manual. If ineffective, alternative analgesics should be used; recommended dosage in the user manual for CYP2D6 normal metabolizers (activity score 1.25–2.25) (Crews et al., 2021)		
CYP3A5*3 (rs776746)	ADR correlation: compared with patients with *CYP3A5* * 1/* 1 and * 1/* 3 genotypes, patients with * 3/* 3 genotypes have an increased risk of adverse events after taking fentanyl (Nakatsu et al., 2024)	Fentanyl Oxycodone	3
	Dose-related: compared with patients with *CYP3A5* * 1/* 1 or * 1/* 3 genotypes, patients with * 3/* 3 genotypes require an increased dose of oxycodone (Naito et al., 2013)		
*ABCB1* (rs1045642)	ADR correlation: Patients with AA genotypes are more likely to experience respiratory depression when using fentanyl than those with AG or GG genotypes (Zhao et al., 2019)	Fentanyl Oxycodone Morphine	3
	Dose-related: Patients with TT genotypes have stronger analgesic effects and lower demand for oxycodone treatment than those with CT or CC genotypes (Lotsch et al., 2009) Patients with TT genotypes have stronger analgesic effects and reduced demand when treated with morphine than those with CT or CC genotypes, but there are opposite conclusions (Campa et al., 2008)		
*OPRM1* (rs1799971)	ADR correlation: Patients with AA genotypes are more likely to experience ADRs, including allergic reactions and respiratory depression, when using oxycodone and morphine than those with AG or GG genotypes (Zheng et al., 2021; Olsen et al., 2019)	Oxycodone Morphine Fentanyl Tramadol Codeine	3
	Dose correlation: Patients with AA genotypes have better analgesic effects on oxycodone, morphine, and tramadol than those with AG or GG genotypes; related to the analgesic effect of fentanyl, but with opposite conclusions (Jones et al., 2019; Takemura et al., 2024)		

Note: The sequence changes in the CYP2D6 * 10 allele are mainly due to rs1065852, whose mutation can lead to a decrease in CYP2D6 enzyme activity; however, if activity scoring is performed, five SNP sites in the table need to be tested.

### 3.2 Patient enrollment status

#### 3.2.1 General information on patients enrolled in the ADR research group

A total of 190 patients were included in the observation subgroup. ADR types specifically included the following: 89 cases of constipation or urinary retention; 75 cases of nausea, vomiting, or abdominal distention; 32 cases of rash or skin itching; 19 cases of chest tightness, wheezing, or suffocation (partial overlap). The specific medications used were as follows: 126 cases of oxycodone hydrochloride sustained-release tablets/capsules, 53 cases of morphine hydrochloride sustained-release tablets/morphine injection, and 11 cases of codeine phosphate tablets. A total of 190 patients were included in the control subgroup, and no ADR occurred. The specific medications used were as follows: 128 cases of oxycodone hydrochloride sustained-release tablets/capsules, 53 cases of morphine hydrochloride sustained-release tablets/morphine injection, and 9 cases of codeine phosphate tablets.

There were no significant differences (*P* > 0.05) in age, gender, body mass index (BMI), initial NRS score, and daily dose of analgesic drugs between the two groups of patients. The baseline was consistent, and the specific data comparison is shown in Table 2.

**TABLE 2:** Comparison of general information on ADR research group-enrolled patients.

Item	Observation subgroup (n = 190)	Control subgroup (n = 190)	t/χ^2^	*P*-value
Age (x̅±s), year	65.1 ± 6.2	64.8 ± 7.1	1.011	0.312
Gender, example	Male 95; female 95	Male 90; female 100	0.169	0.681
BMI (x̅±s, kg/m^2^)	18.5 ± 2.7	18.6 ± 2.6	0.201	0.811
NRS score (x̅±s, point)	6.5 ± 1.1	6.3 ± 1.1	−1.498	0.135
Daily dose (calculated as oral morphine), mg	91.2 ± 45.0	91.6 ± 44.2	0.092	0.927

Note: The measurement data are presented as the mean ± standard deviation.

#### 3.2.2 General information on patients enrolled in the dose demand research group

Due to the small number of patients with daily doses ≥150 mg (calculated as oral morphine) and the need to maintain baseline consistency, especially with similar NRS scores, enrollment was difficult and the sample size was limited. Ultimately, 50 patients were included in the observation subgroup, while 50 patients were included in the control subgroup, and neither group experienced severe ADRs. The following drugs are used: oxycodone hydrochloride sustained-release tablets/capsules, morphine hydrochloride sustained-release tablets, and morphine injection. There were no significant differences in gender, age, BMI value, and initial NRS score between the two groups of patients (*P* > 0.05), and the baseline was consistent. The specific data comparison is shown in Table 3.

**TABLE 3:** Comparison of general information on patients in the dose demand research group.

Item	Observation subgroup (n = 50)	Control subgroup (n = 50)	t/χ^2^	*P*-value
Age (x̅±s), year	62.2 ± 10.1	61.5 ± 9.4	1.621	0.105
Gender, example	Male 26; female 24	Male 25; female 25	0.001	1.001
BMI(x̅±s, kg/m^2^)	17.8 ± 5.2	18.2 ± 3.6	−1.581	0.114
NRS score (x̅±s, point)	7.2 ± 2.1	6.9 ± 2.5	1.127	0.263

Note: The measurement data are presented as the mean ± standard deviation.

### 3.3 Correlation analysis

#### 3.3.1 Correlation analysis between genetic polymorphism and ADR caused by opioid analgesics

Statistical analysis of genotype distribution was performed in two groups of patients in the ADR research group. First, the HWE program was used to detect and analyze whether the genotype distribution conforms to the HWE equilibrium law. The observed values of the two groups of genotype distributions were compared with the expected values, and a chi-square test was performed. The results showed that *P* > 0.05%, indicating that the genotype and allele distributions at each locus conform to genetic equilibrium and are representative of the population. Then, for CYP2D6*10 (rs1065852), *OPRM1* (rs1799971), *ABCB1* (rs1045642), and CYP3A5*3 (rs776746) in both groups, the genotype distribution and allele frequency of the loci were compared. It was found that the AG + GG genotype distribution of *OPRM1* (rs1799971, A>G) was lower and the AA genotype distribution was higher in the observed subgroup (*P* < 0.05) than those in the control subgroup. No distribution differences were observed between the two groups for the remaining genotypes (Table 4). Due to the limited inclusion of cases of codeine (a total of 20 cases), clinical use of compound preparations containing codeine, such as paracetamol dihydrocodeine, which did not meet the inclusion criteria, was excluded. Additionally, both groups were found to be of normal metabolic type, so no statistical analysis was conducted.

**TABLE 4:** Genotype distribution and comparison results with allele frequency of the study population in the ADR research group.

SNP Id	Observation subgroup n = 190 (%)	Control subgroup n = 190 (%)	χ^2^	*P*-value	OR (95% CI)
CYP2D6*10 (rs1065852, C>T)					
CC	86 (45.26)	75 (39.47)			
CT	68 (35.79)	84 (44.21)			
TT	36 (18.95)	31 (16.32)			
CT + TT	104 (54.74)	115 (60.53)	3.160	0.368*	1.049 (0.921–1.195)
Allelic					
C	240 (63.16)	234 (61.58)			
T	140 (36.84)	146 (38.42)	0.202	0.653	1.070 (0.798–1.435)
*OPRM1* (rs1799971, A>G)					
AA	99 (52.11)	72 (37.89)			
AG	66 (34.74)	92 (48.42)			
GG	25 (13.16)	26 (13.68)			
AG + GG	91 (47.90)	118 (62.10)	10.834	0.013*	1.164 (1.021–1.327)
Allelic					
A	264 (69.47)	236 (62.11)			
G	116 (30.53)	144 (37.89)	4.583	0.032	1.389 (1.028–1.876)
*ABCB1*(rs1045642, C>T)					
CC	64 (33.68)	72 (37.89)			
CT	95 (50.00)	90 (47.37)			
TT	31 (16.32)	28 (14.74)			
CT + TT	126 (66.32)	118 (62.11)	1.353	0.717*	0.943 (0.826–1.077)
Allelic					
C	223 (58.68)	234 (61.58)			
T	157 (41.32)	146 (38.42)	1.127	0.288	0.852 (0.634–1.145)
CYP3A5*3 (rs776746, A>G)					
AA	28 (14.74)	22 (11.58)			
AG	60 (31.58)	57 (30.00)			
GG	102 (53.68)	111 (58.42)			
AG + GG	162 (85.26)	168 (88.42)	1.236	0.744*	1.066 (0.911–1.246)
Allelic					
A	116 (30.53)	101 (26.58)			
G	264 (69.47)	279 (73.42)	1.451	0.228	1.214 (0.885–1.664)

Note: * indicates calculations based on grouped phenotypes: CT + TT vs CC and AG + GG vs AA.

Considering the differences and inconsistent research results in the relationship between gene polymorphism and ADR among different opioid analgesics, statistics were conducted according to specific drugs. Among them, statistics were conducted on the most commonly used oxycodone (sustained-release tablets or capsules), and the results showed that there was also a difference in *OPRM1* (rs1799971, A>G) between 126 patients who experienced ADR after using oxycodone and 128 patients who did not experience ADR. In particular, in the observation subgroup, the AA type accounted for 56.35% and the AG + GG type accounted for 43.65%. In the control subgroup, the AA type accounted for 38.28% and the AG + GG type distribution accounted for 61.72%. The proportion of *OPRM1* (rs1799971, A>G) AA type was higher in the experimental subgroup than in the control subgroup (*P* < 0.05) (Table 5).

**TABLE 5:** *OPRM1* genotype comparison results of patients using hydrocodone in the ADR research group.

SNP Id	Observation subgroup n = 126 (%)	Control subgroup n = 128 (%)	χ^2^	*P*-value	OR (95% CI)
AA	71 (56.35)	49 (38.28)			
AG	40 (31.75)	59 (46.09)			
GG	15 (11.90)	20 (15.63)			
AG + GG	55 (43.65)	79 (61.72)	11.000	0.012*	1.234 (1.050–1.451)
Allelic					
A	182 (72.22)	157 (61.33)			
G	70 (27.78)	99 (38.67)	6.789	0.009	1.639 (1.129–2.381)

Note: * indicates calculations based on the AG + GG genotype group compared to AA.

At the same time, the SNPStats program was used to analyze the association between SNP polymorphism and ADR in five genetic models (co-dominant, dominant, recessive, super dominant, and allele models). The model fit was evaluated based on the Akaike Information Criterion (AIC), and the lower the value, the better the model. According to the model results (Table 6), co-dominant, dominant, and allele models all suggest a significant association between *OPRM1* (rs1799971) and the incidence of ADR (*P* < 0.05), with the co-dominant genetic model being the optimal genetic model (*P* = 0.013; AIC = 1885.2), once again proving the correlation between AA type and A-containing alleles in *OPRM1* (rs1799971) and the occurrence of ADR after opioid use.

**TABLE 6:** Genetic model analysis of the correlation between genetic polymorphism and the incidence of ADRs.

SNP Id	Model	Genotype	OR	95% CI	*P*-value	AIC
CYP2D6*10 (rs1065852, C>T)	Codominant	CC/CT/TT	1.05	0.92–1.19	0.368	1890.5
	Dominant	CT/TT vs. CC	1.07	0.80–1.43	0.653	1895.0
	Recessive	TT vs. CC/CT	1.02	0.89–1.16	0.718	1898.2
	Overdominant	CT vs. CC/TT	0.98	0.87–1.11	0.489	1900.3
	Allelic	T vs. C	1.04	0.88–1.22	0.323	1893.6
*OPRM1* (rs1799971, A>G)	Codominant	AA/AG/GG	1.16	1.02–1.33	0.013	1885.2
	Dominant	AG/GG vs. AA	1.28	1.10–1.48	0.025	1891.3
	Recessive	GG vs. AA/AG	1.12	0.97–1.29	0.068	1889.0
	Overdominant	AG vs. AA/GG	1.05	0.92–1.21	0.199	1887.5
	Allelic	G vs. A	1.34	1.10–1.64	0.032	1892.3
*ABCB1*(rs1045642, C>T)	Codominant	CC/CT/TT	0.94	0.83–1.08	0.717	1900.1
	Dominant	CT/TT vs. CC	0.85	0.63–1.15	0.288	1903.5
	Recessive	TT vs. CC/CT	1.09	0.94–1.27	0.412	1904.6
	Overdominant	CT vs. CC/TT	1.01	0.88–1.15	0.602	1902.4
	Allelic	T vs. C	0.98	0.78–1.21	0.492	1901.2
CYP3A5*3 (rs776746, A>G)	Codominant	AA/AG/GG	1.08	0.91–1.25	0.312	1898.2
	Dominant	AG/GG vs. AA	1.05	0.85–1.31	0.444	1899.8
	Recessive	GG vs. AA/AG	1.07	0.89–1.27	0.522	1901.5
	Overdominant	AG vs. AA/GG	1.02	0.82–1.19	0.601	1903.3
	Allelic	G vs. A	1.11	0.94–1.31	0.228	1904.1

#### 3.3.2 Correlation analysis between gene polymorphism and dose demand for opioid analgesics

The dose demand research group analyzed the association between the daily dose of opioid analgesics and gene polymorphism in patients. Based on a daily dose of 150 mg (calculated as oral morphine), 50 patients with a daily dose of ≥150 mg and 50 patients with a daily dose of <150 mg were included. The drugs used were oxycodone sustained-release tablets/capsules and morphine injection/morphine sustained-release tablets. The difference in genotype distribution was statistically analyzed, and the results showed that there was no significant difference in genotype distribution between the observation subgroup (high-dose group) and the control subgroup (low-dose group) (*P* > 0.05) (Table 7).

**TABLE 7:** Genotype distribution and comparison results with the allele frequency of the study population in the dose research group.

SNP Id	Observation subgroup n = 50 (%)	Control subgroup n = 50 (%)	χ^2^	*P*-value	OR (95% CI)
CYP2D6*10 (rs1065852, C>T)					
CC	25 (50.00)	22 (44.00)			
CT	18 (36.00)	20 (40.00)			
TT	7 (14.00)	8 (16.00)			
CT + TT	25 (50.00)	28 (56.00)	0.475	0.924*	1.075 (0.833–1.386)
Allelic					
C	68 (68.00)	64 (64.00)			
T	32 (32.00)	36 (36.00)	0.357	0.55	1.195 (0.665–2.147)
*OPRM1* (rs1799971, A>G)					
AA	30 (60.00)	26 (52.00)			
AG	15 (30.00)	17 (34.00)			
GG	5 (10.00)	7 (14.00)			
AG + GG	20 (40.00)	24 (48.00)	0.997	0.802*	1.118 (0.862–1.449)
Allelic					
A	75 (75.00)	69 (69.00)			
G	25 (25.00)	31 (31.00)	0.627	0.428	1.286 (0.690–2.397)
*ABCB1*(rs1045642, C>T)					
CC	25 (50.00)	20 (40.00)			
CT	20 (40.00)	15 (30.00)			
TT	5 (10.00)	15 (30.00)			
CC + CT	45 (90.00)	35 (70.00)	6.986	0.072*	1.018 (0.806–1.285)
Allelic					
C	70 (70.00)	55 (55.00)			
T	30 (30.00)	45 (45.00)	3.125	0.077	1.690 (0.943–3.029)
CYP3A5*3 (rs776746, A>G)					
AA	8 (16.00)	9 (18.00)			
AG	16 (32.00)	17 (34.00)			
GG	26 (52.00)	24 (48.00)			
AG + GG	42 (42.00)	41 (41.00)	0.176	0.981*	0.958 (0.717–1.281)
Allelic					
A	32 (32.00)	35 (35.00)			
G	68 (68.00)	65 (65.00)	0.202	0.653	0.874 (0.486–1.573)

Note: * indicates calculations based on the following genotype group comparisons: CT + TT vs CC, AG + GG vs AA, and CC + CT vs TT.

## 4 Discussion

The study addresses the issue of individual differences in analgesic efficacy and toxic side effects of opioid analgesics in clinical applications. First, the ADR and efficacy-related genes of opioid analgesics currently confirmed by guidelines, authoritative genetic information database recommendations, and low-bias clinical trials were systematically summarized using evidence-based methods for medical literature retrieval and evaluation, and CYP2D6, CYP3A5*3, ABCB1, and *OPRM1* with strong correlation evaluation and high mutation frequency in the Chinese population were selected as target genes for target population detection. By involving clinically controlled trial protocols, strict inclusion and exclusion criteria, and preliminary estimation of sample size, a total of 480 enrolled patients were divided into two research groups for ADR association evaluation and drug dose association evaluation.

After association analysis and genetic model evaluation, it was found that patients with the *OPRM1* (rs1799971) AA genotype had a higher risk of developing ADRs. *OPRM1* is the main receptor for opioid drugs, and its genetic polymorphism is currently considered the main factor affecting the efficacy of opioid drugs (Agullo et al., 2024). There are many *OPRM1* mutation sites, among which rs1799971 is a common SNP. Many clinical studies have shown that this site mutation significantly affects the clinical efficacy of opioid drugs. It is believed that patients with AA genotypes have better analgesic effects on oxycodone, morphine, and tramadol than those with AG or GG genotypes, and the specific mechanism may be related to higher target sensitivity (Chidambaran et al., 2015). In terms of ADR incidence, some studies suggest that patients with AA genotypes are more likely to experience ADR, including allergic reactions and respiratory depression, when using oxycodone or morphine than those with AG or GG genotypes. In the ADR research group, it was found that the distribution of AA types and A allele genes was significantly higher in the observation subgroup (patients experiencing ADR) than in the control subgroup (patients who did not experience ADR), which is consistent with the literature evaluation results and can be mutually verified; patients with AA genotypes should pay more attention to the adverse drug reactions of oxycodone and morphine. The dosage should begin at a low level and be closely monitored for any allergic reactions, especially signs of respiratory depression. Unfortunately, no differentially expressed genes were found in the medication demand evaluation group, which may be related to the limited sample size. In addition, existing results also suggest the need to pay attention to the *ABCB1* gene. Under the current sample size, the *ABCB1* (rs1045642) CC + CT ratio in the observed subgroup tends to be higher than that in the control subgroup. Studies have shown that the polymorphism of the *ABCB1* gene, especially rs1045642, can affect the demand for opioid analgesics. Patients with TT genotypes may have a lower demand for oxycodone dosage (PharmGKB grade: 3) than those with CT and CC genotypes. Studies suggest that the main reason is related to changes in P-gp protein expression or transport function caused by gene polymorphism. In addition, P-gp can also limit the distribution of opioid analgesics in the brain. Differences in its expression levels can lead to differences in the concentration of opioid analgesics distributed in the brain, especially in the cortex, hippocampus, and vascular areas, resulting in the compression of opioid analgesics outside the central nervous system, leading to tolerance to opioid analgesics, and requiring higher doses to achieve analgesic effects (Qin et al., 2024). Some studies have also found a close relationship between the *ABCB1* gene polymorphism and pain perception in cancer patients. There are differences in β-endorphin levels among patients with different genotypes of *ABCB1* rs1045642, which leads to differences in the degree of pain in patients (Ofoegbu and Ettienne, 2021). In addition, the relationship between CYP2D6 metabolism and the dosage and toxicity of codeine and tramadol is clear, and it is recommended for CPIC guideline grade A and PharmGKB grade 1A. Therefore, although this study was limited by the small sample size of patients using codeine and no correlation could be evaluated, it is still recommended to assess the metabolic activity types of CYP2D6 when prescribing codeine or tramadol in clinical practice.

This study also has certain limitations. First, the included detection sites are limited. Currently, its value mainly lies in serving as a reference model for research, and there is still a long way to go before a large-scale gene locus database can be established. Second, the sample size is not yet sufficient to fully demonstrate the overall genetic diversity of the population and is also influenced by complex variables such as cancer type, cancer stage, and differences between drug types. At the same time, the tolerance of different individuals to pain is also an important factor affecting the accuracy of research results. Cancer pain patients exhibit significant variability in pain sensitivity. Therefore, more rigorous clinical trial settings are needed for future research to exclude heterogeneity effects and further evaluate the association between gene polymorphism and opioid analgesics.

## 5 Conclusion

Patients with *OPRM1* (rs1799971) AA genotypes should pay more attention to the ADRs of oxycodone and morphine. The dosage should begin at a low level and be closely monitored for any allergic reactions, especially signs of respiratory depression. The association between drug dose requirements and SNPs still needs to be evaluated in larger cohorts. Meanwhile, particular attention should be paid to the *ABCB*1 gene.

## Data Availability

The datasets presented in this study can be found in online repositories. The names of the repository/repositories and accession number(s) can be found below: https://www.ncbi.nlm.nih.gov/snp/, rs1799971.
